# How hazard ratios can mislead and why it matters in practice

**DOI:** 10.1007/s10654-025-01250-9

**Published:** 2025-06-27

**Authors:** Elise Dumas, Mats J. Stensrud

**Affiliations:** Institute of Mathematics, Ecole Polytechnique Fédérale de Lausanne, Station 8, 1015 Lausanne, Switzerland

## Abstract

Hazard ratios are routinely reported as effect measures in clinical trials and observational studies. However, many methodological works have raised concerns about the interpretation of hazard ratios as causal effects. These concerns are often related to three points: (i) depletion of susceptible individuals leads to selection bias and complicates the causal interpretation of the hazard ratio, (ii) the hazard ratio is not collapsible, and (iii) the conventional proportional hazards assumption rarely holds in medical studies. We discuss the relation between these three points. We ground our presentation on an example about effect of endocrine therapy in reducing the risk of recurrence or death in a population of patients with breast cancer. We also describe why survival curves and risk differences do not exhibit any of the undesirable properties of hazard ratios.

**Supplementary Information:**

The online version contains supplementary material available at 10.1007/s10654-025-01250-9.

## Introduction

There is a growing literature on the limitations of the hazard ratio as a causal measure of treatment effects. Criticisms of the use of hazard ratios focus on three of its properties: its built-in selection bias [1–3], its non-collapsibility [4–6], and violations of the proportional hazards assumption [2, 7, 8]. While some authors have argued that these issues compromise the interpretability of the hazard ratio, others have argued for the continued use of the hazard ratio as a valid and informative summary measure [9–13]. Meanwhile, hazard ratios remain widely used in clinical research, like in oncology [14, 15], cardiology [16], vaccine studies [17, 18], and neurology [19]. In this work, we illustrate the three key concerns around hazard ratios, and why they matter in practice, through two clinical trial examples concerning endocrine therapy and breast cancer. We further discuss how hazard ratios are related to risk ratios. Finally, we describe why causal estimands based on survival curves do not exhibit the limitations of hazard ratios. We also give a more general and technical elaboration in the Appendix.

## Hazard ratios in practice: two trials on endocrine therapy

Breast cancer is a heterogeneous disease, commonly classified by hormone-receptor status. Hormone-receptor negative tumors are more aggressive and associated with higher risks of recurrence than hormone-receptor positive tumors [20, 21]. Endocrine therapy, developed to block hormone-driven tumor growth, is effective in reducing the risk of disease recurrence and death of hormone-receptor positive tumors, but not in hormone-receptor negative tumors [22]. To illustrate the properties of hazard ratios, we present two hypothetical trials for endocrine therapy. Although based on synthetic data, the survival curves were designed to reflect realistic clinical scenarios.

### Trial one

Consider a clinical trial randomly assigning patients with early-stage breast cancer to endocrine therapy (treatment group, $T=1$) *versus* no treatment (control group, $T=0$). The primary endpoint is disease-free survival at five years, which we will refer to simply as survival. Suppose that 50% of the patients have a hormone-receptor positive disease ($X=0$) and 50% have a hormone-receptor negative disease ($X=1$).^1^

**Fig. 1:**
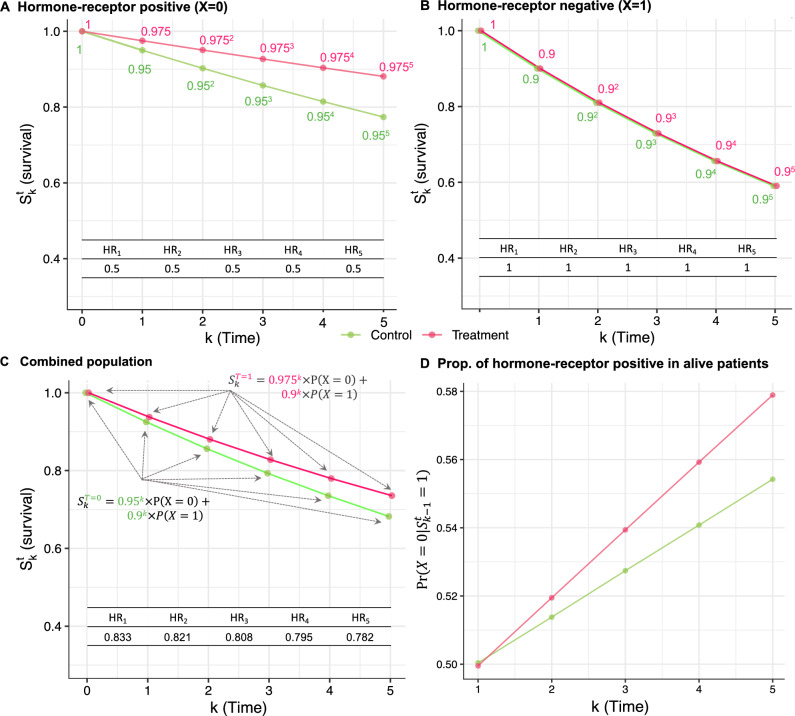
Results for the first trial. **A**–**C** Survival probabilities and hazard ratios over the first five time points (years) in the treatment group (endocrine therapy, in pink) and control group (no treatment, in green) among **A** patients with hormone-receptor positive disease; **B** patients with hormone-receptor negative disease; and **C** the combined population. **D** Proportion of patients with hormone-receptor positive disease among patients still alive in the treatment and control groups. Abbreviations: HR, hazard ratio; Prop., proportion

For treatment $t \in \{0,1\}$, define the survival function ($S_k^t$) as the probability of being alive up to time *k* under treatment *t*; the hazard ($h_k^t$) as the probability^2^ of death at *k* given survival up to time $k-1$ under treatment *t*; and the hazard ratio ($HR_k$) as the ratio of hazard at time *k* under treatment and control. In the hormone-receptor positive subgroup, we assume that the hazard under control is equal to 0.05 and the hazard ratio is equal to 0.5 at all time points (protective effect of treatment, Fig. 1A). In the hormone-receptor negative subgroup, we assume that the hazard under control is equal to 0.1 and the hazard ratio is equal to 1 at all time points (no treatment effect, Fig. 1B).

**The hazard ratio is usually time-varying**. While the hazard ratio, by construction, is constant over time in both hormone-receptor positive and hormone-receptor negative disease subgroups, the hazard ratio decreases over time in the combined population (Fig. 1C). Therefore, the hazards are not proportional in the combined population; that is, the *proportional hazard assumption* does not hold marginally. More generally, the proportional hazards assumption is implausible in populations that include individuals with different susceptibilities to death, because of different treatment effects, frailty, or both [7].

**The hazard ratio is not strictly collapsible.** Survival at time *k* in the combined population can be written as a weighted average of survival proportions in the two subgroups, with weights being the proportion of patients in the two subgroups (Fig. 1C). We say that the survival function is *strictly collapsible* [24]. In contrast, the hazard ratio does not have this property. For example, at the first time point, $0.5 \times Pr(X=0) + 1 \times Pr(X=1) = 0.75 \ne 0.833 = HR_1$.

**The hazard ratio is prone to selection bias.** At baseline, the proportion of patients with hormone-receptor positive tumor is the same in the control and in the treatment group because randomization ensures covariate balancing in expectation (Fig. 1D). Yet, the proportion of patients with hormone-receptor positive disease increases over time in both treatment groups. This is expected because events (recurrences or deaths) occur more often and earlier in the hormone-receptor negative subgroup compared with the hormone-receptor positive subgroup. This phenomenon is broadly called *depletion of the susceptibles* [25]. Furthermore, the proportion of patients with hormone-receptor positive disease increases faster in the treatment group compared to the control group: the mechanisms of depletion of the susceptibles is *differential*. Because the frequency of hormone-receptor positive disease differs in the control and treatment groups after time one, the hazard ratio is said to suffer from *selection bias* [2, 3] (also called *depletion-of-susceptibles bias* [26, 27]). However, to be precise, bias should always be defined with respect to a specific estimand. Implicitly, the term selection bias refers to causal effects, that is, contrasts of potential outcomes within the same subpopulation of individuals. Due to the differential depletion of susceptibles over time in the two treatment arms, the hazard ratio cannot be straightforwardly interpreted as a causal effect.

**The hazard ratio differs from the risk ratio.** Except at the first time point, the hazard ratio cannot be interpreted as the ratio of the risk of event in the treatment and control group [28, 29]. For example, at the fifth time point in the hormone-receptor positive subgroup, the ratio of the risk of events is $(1-0.975^5)/(1-0.95^5) \approx 0.53 \ne 0.5 = HR_5$ (Fig. 1A). The discrepancy may appear small in this case, but it would be substantially more pronounced with longer follow-up or a larger proportion of events.

### Trial two

**Fig. 2:**
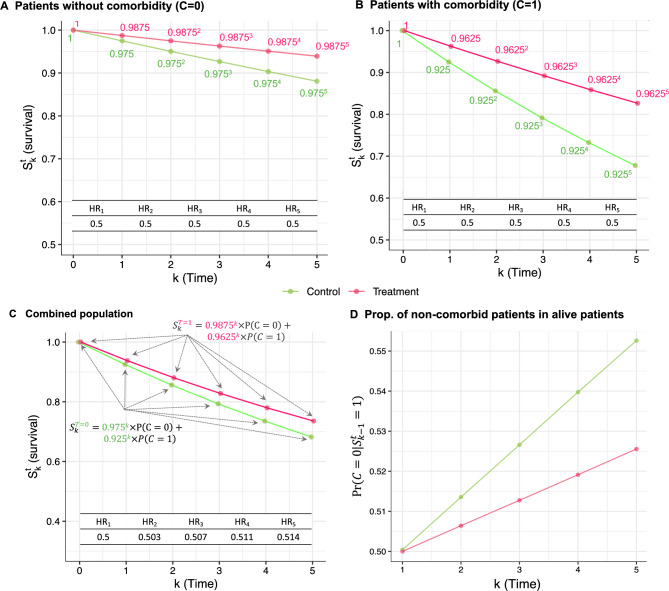
Results for the second trial. **A**–**C** Survival probabilities and hazard ratios over the first five time points (years) in the treatment group (endocrine therapy, in pink) and control group (no treatment, in green) among **A** patients without comorbidity; **B** patients with comorbidity; and **C** the combined population. **D** Proportion of patients without comorbidity among patients still alive in the treatment and control groups. Abbreviations: HR, hazard ratio; Prop., proportion

Consider a second randomized clinical trial including only patients with hormone-receptor positive disease. Imagine that the population can be divided into two subgroups: patients without comorbidity at baseline ($C=0$), and patients with at least one comorbidity at baseline ($C=1$). Suppose that the hazard ratio of endocrine therapy (treatment) *versus* control is constant equal to 0.5 for both subgroups, *i.e*, there is no heterogeneity at the hazard ratio scale between the subgroups. Suppose further that the hazard under no treatment is constant in both subgroups, and equals 0.025 in the absence of comorbidities and 0.075 otherwise (Fig. 2A, B).

**The hazard ratio lies outside of the subgroup hazard ratios**. Even in this setting where the hazard ratios are equal to 0.5 in the two subgroups, the hazard ratio in the combined population differs from 0.5 after time 2 (Fig. 2C). This shows that not only the hazard ratio is not strictly collapsible, but the hazard ratio also cannot be written as a weighted average of the subgroup hazard ratios. We say that the hazard ratio is not even *collapsible* [5]. The example also shows that the hazard ratio in the combined population is not always between the minimum and the maximum of the subgroup hazard ratios.

**The hazard ratio is prone to selection bias even in the absence of treatment effect heterogeneity**. The proportion of patients without comorbidity ($C=0$) increases more rapidly in the control group than in the treatment group (Fig. 2D) despite the treatment exerting the same effect across subgroups at the hazard ratio scale. This pattern highlights that the selection bias inherent to hazard ratios is not exclusively driven by treatment effect heterogeneity (on the hazard ratio scale), and can also arise from differences in underlying frailty within the population.

**The hazard ratio is a relative measure, but not a relative risk.** Even if the hazard ratio is the same in the two subgroups, the difference in survival probabilities between treatment and control at five years differs substantially in the two subgroups due to the different susceptibility to recurrence and death under control. For example, at the fifth time point, the survival difference between the treatment and control group is $0.9875^5 - 0.975^5 \approx 5.8\%$ without comorbidity, compared to $0.9625^5-0.925^5 \approx 14.9\%$ with comorbidity. This illustrates the differences in interpretation of relative measures, e.g. a hazard ratio, *versus* absolute measures, e.g. a survival difference [30].

## Hazard ratios are not risk ratios

Beyond the statistical concerns outlined above, hazard ratios are inherently difficult to understand and are often misinterpreted as risk ratios [28, 29]. For example, stating that “endocrine therapy reduces the risk of recurrence or death by 50% in patients with HR-positive disease” based on a hazard ratio of 0.5 is potentially misleading, as hazard ratios reflect instantaneous event rates, not cumulative risks. In clinical practice, however, cumulative risk or survival estimates reported at specific follow-up times are routinely used. For example, five-year survival is commonly communicated to cancer patients.

Furthermore, because hazard ratios are measured on a relative scale, their interpretation is sensitive to baseline risks in the control group [30]. We therefore need to interpret the magnitude of the effect with care, as it may be substantially smaller when expressed on other scales [31]. Patients and clinicians often find absolute measures of risk more interpretable [32] and there are recommendations to avoid reporting relative effect measures alone [33]. However, presenting absolute hazard differences to complement hazard ratios would require specifying or estimating the baseline hazard function, which is typically left unspecified in the semi-parametric Cox proportional hazards model. Even when reported, absolute hazard estimates are also hard to interpret causally [4].

**Fig. 3:**
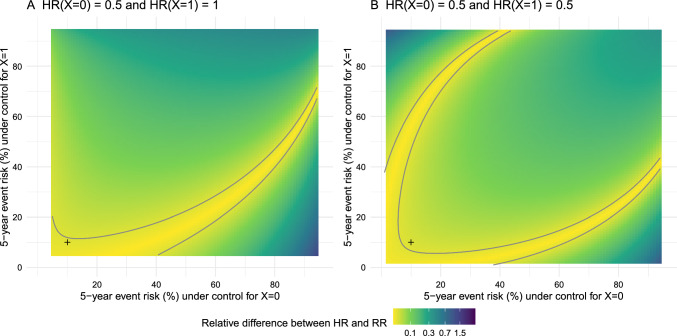
Relative difference between the hazard ratio and the risk ratio for different values of the subgroup risk of events at five years under **A** the first trial, and **B** the second trial. The contour line for 2% relative difference is plotted in gray. The cross point represents a 10% risk of event at five years in both subgroups. We assumed that the hazard under control and the hazard ratio was constant over time in both subgroups and that $Pr(X=0) = 0.5$. Abbreviations: HR, hazard ratio; RR, risk ratio

If the probability of experiencing the event is low, the hazard function approximately equals the risk function, so that the hazard ratio approximately equals the risk ratio. This justifies the use of hazard ratios as approximations of risk ratios when outcome events are rare. The risk ratio is not prone to the built-in selection bias of the hazard ratio and is collapsible. However, determining the threshold of risk below which the risk ratio can be reasonably approximated by the hazard ratio is not straightforward and is context-dependent. As an illustration, if we accept a 2% relative difference between the hazard ratio and the risk ratio, we would consider 5-year risks of event of 10% in the two subgroups to be acceptable in the scenario of the first trial but not of the second trial (Fig. 3).

Finally, if the risk ratio is the measure of interest, there is no need to approximate it using the hazard ratio, since risk ratios can be directly estimated by alternative methods, such as from survival curves, without relying on the rare event assumption.

## Survival functions do not suffer from any of the problems concerning hazard ratios

Survival curves, and differences in survival probability at specific points in time, are not prone to any of the three limitations of hazard ratios described above. First, effects on the survival scale do not require conditioning on survival up to a certain time point. Thus, they are not subject to the built-in selection bias of hazard ratio. Second, survival differences are strictly collapsible (and survival ratios are collapsible). Third, the estimation of survival curves can be done non-parametrically in randomized clinical trials, e.g., using the Kaplan-Meier estimator [34], thereby not relying on the proportional hazard assumption. In observational studies, where adjustment for baseline confounders is needed, adjusted parametric survival curves can be reconstructed from any hazard model that does not assume proportional hazards [2]. Alternatively, adjusted survival curves can be estimated from inverse-probability of treatment weighted [35] or doubly robust [36] procedures. In contrast, the non-collapsibility of hazard ratios makes estimates from classical observational studies – that included other covariates in the Cox proportional hazard model to adjust for confounding – incomparable with hazard ratios from randomized clinical trials – that did not include other covariates in the Cox model.

Unlike hazard ratios, we regard effects on the survival scale as more aligned with our plain language and intuitive notions of treatment effects. This is important for communication and decision making. For example, without any consideration of hazard ratios, we can answer questions such as: does the treatment reduce the risk of death at five years of follow-up? What are the effects after one year? Does the effect on survival after five years differ between groups?

## Conclusion

Hazard ratios suffer from limitations that complicate their interpretation, such as the built-in selection bias, non-collapsibility, and reliance on the often implausible proportional hazards assumption. Informally, the hazard ratio compares the probability, or rate in continuous time, of experiencing the event at a given time among individuals who have survived up to that point. Conditioning on being alive can induce selection bias, as the characteristics of the patients at risk evolve differently in the treatment and control groups [2, 3, 37–39]. The non-collapsibility of hazard ratios implies that the marginal hazard ratio generally cannot be expressed as a weighted average of subgroup-specific hazard ratios, even under randomization, and may lie outside the range of those subgroup effects [4–6, 40–42]. Finally, the proportional hazards assumption, central to the Cox model [43], requires the hazard ratio to be constant over time. This assumption is rarely satisfied, even if the treatment effect is constant in all population subgroups [7, 8]. All three concerns relate to differential depletion-of-the-susceptibles over time. Therefore, hazard ratios may deviate substantially from effect measures on the survival or risk scale outside of overly simplified scenarios when there is no treatment effect, no heterogeneity in disease risk (frailty) and no effect modification at the hazard ratio scale, or when highly specific (and miraculous) cancellations occur across time and subgroups. Further theoretical support for these claims is provided in the Appendix, where we also respond more directly to recent arguments about the appropriateness of hazard ratios [13].

By contrast, effect measures expressed on the survival or risk scale are directly interpretable. They often align more closely with how outcomes are understood and communicated in clinical practice, using plain language. As a result, such measures are arguably more informative for guiding clinical decision-making.

## Supplementary Information

Below is the link to the electronic supplementary material.

Supplementary file 1 (pdf 222 KB)

## Footnotes

1 In the general population, we would rather expect 80% of hormone-receptor positive diseases and 20% of hormone-receptor negative diseases [23], but assuming equal proportions simplifies calculations and intuitions, while the broad conclusions remain the same.

2 Formally, the hazard represents a probability in discrete time, but a rate in continuous time. Throughout this article, we treat time as discrete for ease of exposition.
