# Supplementary material for: How hazard ratios can mislead and why it matters in practice
Source: Eur J Epidemiol. 2025 Jun 27;40(6):603–9. doi: 10.1007/s10654-025-01250-9 (PMC12263472; doi:10.1007/s10654-025-01250-9)
Supplement: Supplementary file 1 — (pdf 222 KB) [file 10654_2025_1250_MOESM1_ESM.pdf]

Appendix
How hazard ratios can mislead and why it matters in practice

Elise Dumas *[1] and Mats J. Stensrud †[1]

[1]Institute of Mathematics, Ecole Polytechnique Fédérale de Lausanne, Station 8, 1015 Lausanne, Switzerland

A.1 Characteristics of hazard ratios in a general setting

We discuss the built-in selection bias of the hazard ratio, the non-collapsibility of the hazard ratio, and the plausibility of the proportional hazard assumption. We explain when these concerns are important and when they are negligible. We also explain how the concerns relate to the same mechanism of susceptible individuals being selected differently in the treatment and control group.

Setting. Suppose that we want to estimate the effect of a binary treatment T on a time-to-event outcome Y (*e.g.* death). Suppose further that the population can be divided into two subgroups, defined as $X = 0$ or $X = 1$. The treatment T might have heterogeneous effects in the subgroups defined by X. Our arguments hold regardless of whether X is measured.

To focus on the main points, we consider a perfectly executed clinical trial where T is randomly assigned. We assume that patients fully adhere to the treatment regime they were assigned to, and that there is no losses-to-follow-up. We will treat time as discrete and assume that the outcome is measured at regular time points $k \in 1, ..., K$ where K is the time horizon. Results analogous to ours will hold in the continuous time case. Furthermore, even if the underlying time scale is continuous, time in clinical studies is practically measured in discrete intervals (days, months, trimesters,...).

We use potential outcome notations to denote counterfactual quantities: Y_k^1 (respectively Y_k^0) is the value of the outcome at time point k that would be observed for an individual if, potentially contrary to the fact, treatment $T = 1$ (respectively $T = 0$) was assigned. We assume the classical causal assumptions of consistency, positivity, and exchangeability.

Survival, hazard, and hazard ratio. For $t \in 0, 1$ and $k \in 1, \ldots, K$, we denote by

$$h_k^t := Pr(Y_k^t = 1 | Y_{k-1}^t = 0)$$

*elise.dumas@epfl.ch, ORCID : 0000-0002-7877-719X
†mats.stensrud@epfl.ch, ORCID : 0000-0001-9641-1936

the counterfactual (discrete) hazard under treatment $T = t$, *i.e.* the probability of experiencing the outcome at time k given that the outcome was not experienced before.[1] The counterfactual survival function S_k^t is the probability of not experiencing the outcome before k under treatment assignment $T = t$,

$$S_k^t = Pr(Y_k^t = 0).$$

The counterfactual hazard ratio of treatment *versus* control at time point k is defined as

$$HR_k = \frac{h_k^1}{h_k^0}.$$

Under the assumptions of consistency, positivity, and exchangeability, the counterfactual hazard ratio is identified by the ratio of the observed hazards [1],

$$HR_k = \frac{Pr(Y_k = 1|Y_{k-1} = 0, T = 1)}{Pr(Y_k = 1|Y_{k-1} = 0, T = 0)}.$$

Similar definitions can be given to the hazard function, survival function, and hazard ratio in the subgroups $X = 0$ and $X = 1$. We will denote subgroup measures with parentheses. For example, the hazard at time k under treatment t in the subgroup $X = 1$ is

$$h_k^t(X = 1) := Pr(Y_k^t = 1|Y_{k-1}^t = 0, X = 1).$$

Built-in selection bias. Because counterfactual hazards are defined by conditioning on previous survival, the hazard ratio compares the risk of death at a given time among patients still alive under treatment and among patients still alive under control. Therefore, a hazard ratio different from one at time k may be attributable to differences in the population's characteristics over time, rather than to an instantaneous treatment effect. This can happen either because the treatment has differential effect in patients with different characteristics, or because patients with different characteristics have different risk of death under control. The so-called built-in selection bias of hazard ratio was discussed by Robins in 1986 [2] and Hernán in 2010 [3], and further extensively discussed by other authors [4, 5, 6, 7].

To fix ideas, suppose that the binary covariate X is associated with experiencing the outcome. At baseline, due to randomization, the distribution of X is balanced in the treatment and control group, *i.e.* $Pr(X = 0|T = 0) = Pr(X = 0|T = 1)$. At future time points, we can show that the distribution of X remains balanced in the treatment and control group if and only if

$$\forall k \geq 1, \frac{1 - HR_k(X = 1) \cdot h_k^0(X = 1)}{1 - HR_k(X = 0) \cdot h_k^0(X = 0)} = \frac{1 - h_k^0(X = 1)}{1 - h_k^0(X = 0)}. \quad (1)$$

The proof is given in Appendix A.2. The equality in (1) holds trivially if the treatment has no effect on the hazard ratio scale in both subgroups at all times; that is, if $\forall k, HR_k(X = 1) = 1 = HR_k(X = 0)$. Alternatively, the equality holds if both (i) $HR_k(X = 1) = HR_k(X = 0)$ and (ii) $h_k^0(X = 0) = h_k^0(X = 1)$, in which case the control and treated

[1] For $k = 0$, we fix $Y_{-1}^t \equiv 0$ almost surely, so that $h_1^t = Pr(Y_1^t = 1)$

survival curves are identical in the two subgroups. In all other cases, equality (1) is unlikely to occur; it can miraculously hold in case of some perfect cancellations of selection bias and effect heterogeneity at all time points, which is highly implausible. Thus, we will expect that the hazard ratio is affected by differential selection of X except if either the treatment has no effect, the variable X is irrelevant for the outcome under both treatment and control, or under an implausible cancellation.

Some authors have used numerical simulations to demonstrate the existence of the built-in selection bias of hazard ratios [4, 7]. In particular, Stensrud *et al* [7] tailored a specific data-generating mechanism to show that, in principle, selection bias can be quite dramatic. The proposed data-generating mechanism was not intended to be plausible, much like any other mathematical counterexample. Instead, it was intended to prove a point: time-varying hazard ratios are generally hard to interpret causally.

On the other hand, attempting to show through simulations the plausible magnitude of bias, as suggested by Abrahamowicz *et al* [8], is a difficult task. One problem is that postulating explicit data-generating mechanisms involves not only specifying known unknowns, but also making claims about unknown unknowns. In particular, fully specifying the mechanisms underlying disease susceptibility and treatment effect heterogeneity, at the hazard scale, along with all the true time-varying effects of treatment is hardly something that humans, even experts, can make strong claims about, at least if they adhere to scientific principles of empirical falsifiability [9]. Moreover, without formally defining causal estimands of interest, along with identification assumptions, it is difficult to interpret claims about bias in a rigorous manner. However, the daunting task of causal interpretation of (time-varying) hazard ratios can be easily avoided. As emphasized in the main text, we advocate for reporting effect measures with a clear causal interpretation that does not rely on the interpretation of potentially time-varying hazard ratios. This view is coherent with existing work in the causal inference literature [2, 3, 7, 10, 11].

Non-collapsibility. Many population causal effects, like a risk or survival difference, can be expressed as a (weighted) average of subgroup effects. However, not all measures of causal effects respect this subgroup decomposition property. Population effects that can be written as a weighted average of subgroup effects are said to be *collapsible* [12]. Furthermore, if the weights are equal to the population's proportion, the measure is said to be *strictly collapsible*. The collapsibility of standard measures has been extensively discussed previously [12, 13, 14, 15, 16, 17].

Hazard ratios are neither strictly collapsible, nor collapsible. We have already demonstrated this in the two trials of endocrine therapy and breast cancer. Here we elaborate by considering the population hazard ratio at time k as a function of the subgroup hazard ratios,

$$HR_k = w_k(X=0) \cdot HR_k(X=0) + w_k(X=1) \cdot HR_k(X=1),$$

with

$$w_k(X=0) = \frac{h_k^0(X=0) \cdot Pr(X=0|Y_{k-1}^1=0)}{h_k^0}$$

and

$$w_k(X=1) = \frac{h_k^0(X=1) \cdot Pr(X=1|Y_{k-1}^1=0)}{h_k^0}.$$

See Appendix A.3. for a proof. The weights $w_k(X=0)$ and $w_k(X=1)$ are positive but generally do not sum to 1.

At the first time point, the weights reduce to $w_1(X=0) = \frac{Pr(Y_1^0=1|X=0)\cdot Pr(X=0)}{Pr(Y_1^0=1)}$ and $w_1(X=1) = \frac{Pr(Y_1^0=1|X=1)\cdot Pr(X=1)}{Pr(Y_1^0=1)}$, thereby summing to 1. However, the weights $w_1(X=0)$ and $w_1(X=1)$ are equal to the subgroup proportions $Pr(X=0)$ and $Pr(X=1)$ if and only if the counterfactual hazards under control are equal in the two subgroups. Indeed, at the first time point, the hazard ratio is equivalent to a risk ratio, which measure has been shown to be collapsible but not strictly collapsible [13].

At further time points, the weights w_k sum to 1 if and only if:

$$\left(Pr(X=0|Y_{k-1}^1=0) - Pr(X=0|Y_{k-1}^0=0)\right)\cdot\left(h_k^0(X=1) - h_k^0(X=0)\right) = 0. \quad (2)$$

See Appendix A.3. for a proof. Equation (2) holds if either

$$Pr(X=0|Y_{k-1}^0=0) = Pr(X=0|Y_{k-1}^1=0),$$

that is, if the distribution of X remains the same in the treated and control group, or if

$$h_k^0(X=0) = h_k^0(X=1),$$

that is, if the counterfactual hazards under control are equal in the two subgroups. In the latter case, the hazard ratio at time point k is collapsible with weights being the actual proportion of the subgroups in the population still alive under treatment. Note that the weights will vary with time in general.

Proportional hazard assumption. A common practice to estimate the hazard ratio of treatment *versus* control in a randomized clinical trial is to fit a Cox proportional hazards model [18]. The Cox proportional hazards model owes much of its popularity to its semiparametric structure, where the baseline hazard function (continuous analogue of h_k^0 with our notations) is treated as nuisance and left unspecified. The model is straightforward to implement using widely available statistical software, and standard routines provide point estimates, confidence intervals, and hypothesis tests with minimal user input. The procedure also allows the treatment effect to be summarized in a single parameter, making it straightforward to report results.

However, the Cox proportional hazards model relies on the assumption of proportional hazards, which states that the hazard ratio is constant over time. Previous works have warned against the implausibility of this assumption [10, 11]. Indeed, the proportional hazard assumption might be violated if treatment effect changes over time, but also if the distribution of susceptibility changes over time in the population. In particular, as we described in the second trial, the hazard ratio is expected to vary over time in the combined population, even if the hazard ratio in the two subgroups are constant and equal one with each other.

To be explicit, we rewrite the hazard ratio,

$$HR_k = \frac{E\left[HR_k(X)h_k^0(X)|Y_{k-1}^1=0\right]}{E\left[h_k^0(X)|Y_{k-1}^0=0\right]},$$

where the expectation is over X. From this expression, we see that, even if $\forall k, HR_k(X = 1) = HR_k(X = 0) =: HR_{\text{subgroup}}$,

$$HR_k = HR_{\text{subgroup}} \cdot \frac{E\left[h_k^0(X)|Y_{k-1}^1 = 0\right]}{E\left[h_k^0(X)|Y_{k-1}^0 = 0\right]}$$

is still expected to vary over time. The proportionality assumption holds if the distribution of X remains the same in the treated and control group, that is, if for all k, $Pr(X = 0|Y_{k-1}^1 = 0) = Pr(X = 0|Y_{k-1}^0 = 0)$. The hazard ratio might also be constant in this case if $h_k^0(X = 0) = h_k^0(X = 1)$, but this would also imply that $Pr(X = 0|Y_{k-1}^1 = 0) = Pr(X = 0|Y_{k-1}^0 = 0)$, as we already assumed that $HR_k(X = 0) = HR_k(X = 1)$.

In the more general case where the hazard ratios are varying across subgroups or time, the hazard ratio in the combined population might still be constant, albeit under some perfect, implausible cancellations of treatment effects and susceptibility across the two subgroups at all times, ensuring that $E\left[HR_k(X)h_k^0(X)|Y_{k-1}^1 = 0\right]$ evolves proportionally to $E\left[h_k^0(X)|Y_{k-1}^0 = 0\right]$. This assumption is heroic and does not correspond to any clinically imaginable scenario. Furthermore, even if it holds, the constant hazard ratio would not have an unambiguous causal interpretation because its magnitude would depend on both the true causal treatment effects and selection bias over time.

A.2 Proofs for built-in selection bias

First note that the counterfactual survival function can be expressed as a function of the counterfactual hazard as follows:

$$S_k^t = \prod_{i=1}^{k} \left(1 - h_i^t\right).$$

Then, for $t \in 0, 1$ and $k \geq 1$, remark that

$$Pr\left(X = 0 | Y_k^t = 0\right) = \frac{S_k^t(X=0)Pr(X=0)}{S_k^t(X=0) \cdot Pr(X=0) + S_k^t(X=1) \cdot Pr(X=1)}$$

by Bayes. Thus, $Pr\left(X = 0 | Y_k^0 = 0\right) = Pr\left(X = 0 | Y_k^1 = 0\right) \forall k \geq 1$ if and only if

$$\frac{S_k^0(X=0)}{S_k^0(X=0) \cdot Pr(X=0) + S_k^0(X=1) \cdot Pr(X=1)} = \frac{S_k^1(X=0)}{S_k^1(X=0) \cdot Pr(X=0) + S_k^1(X=1) \cdot Pr(X=1)}$$

$$\Leftrightarrow \frac{S_k^0(X=0)}{S_k^1(X=0)} = \frac{S_k^0(X=0) \cdot Pr(X=0) + S_k^0(X=1) \cdot Pr(X=1)}{S_k^1(X=0) \cdot Pr(X=0) + S_k^1(X=1) \cdot Pr(X=1)}$$

$$\Leftrightarrow \frac{S_k^0(X=0)}{S_k^1(X=0)} = \frac{\frac{S_k^0(X=0)}{S_k^1(X=0)} + \frac{S_k^0(X=1)}{S_k^1(X=0)} \cdot \frac{Pr(X=1)}{Pr(X=0)}}{1 + \frac{S_k^1(X=1)}{S_k^1(X=0)} \cdot \frac{Pr(X=1)}{Pr(X=0)}}$$

$$\Leftrightarrow \frac{S_k^0(X=0)}{S_k^1(X=0)} \cdot \frac{S_k^1(X=1)}{S_k^1(X=0)} = \frac{S_k^0(X=1)}{S_k^1(X=0)}$$

$$\Leftrightarrow \frac{S_k^0(X=0) \cdot S_k^1(X=1)}{S_k^1(X=0)} = S_k^0(X=1)$$

$$\Leftrightarrow \frac{S_k^1(X=0)}{S_k^0(X=0)} = \frac{S_k^1(X=1)}{S_k^0(X=1)}$$

$$\Leftrightarrow \prod_{i=1}^{k} \frac{1 - HR_i(X=0) \cdot h_i^0(X=0)}{1 - h_i^0(X=0)} = \prod_{i=1}^{k} \frac{1 - HR_i(X=1) \cdot h_i^0(X=1)}{1 - h_i^0(X=1)}$$

$$\Leftrightarrow \prod_{i=1}^{k} \frac{1 - HR_i(X=1) \cdot h_i^0(X=1)}{1 - HR_i(X=0) \cdot h_i^0(X=0)} \cdot \frac{1 - h_i^0(X=0)}{1 - h_i^0(X=1)} = 1, \tag{3}$$

We assumed that $\forall k, S_k^0, S_k^1 \neq 0$ (the survival curves do not reach 0 within the end of the trial), and that $0 < Pr(X=1) < 1$. Next, as (3) needs to be true at every k, we can show by induction that

$$\forall k \geq 1,\ Pr\left(X = 0 | Y_k^0 = 0\right) = Pr\left(X = 0 | Y_k^1 = 0\right)$$

$$\Leftrightarrow \forall k \geq 1, \frac{1 - HR_k(X=1) \cdot h_k^0(X=1)}{1 - HR_k(X=0) \cdot h_k^0(X=0)} \cdot \frac{1 - h_k^0(X=0)}{1 - h_k^0(X=1)} = 1.$$

A.3 Proofs for non-collapsibility

First, notice that

$$
\begin{aligned}
HR_k &= \frac{h_k^1}{h_k^0} \\
&= \frac{h_k^1(X=0)Pr(X=0|Y_{k-1}^1=0) + h_k^1(X=1)Pr(X=1|Y_{k-1}^1=0)}{h_k^0} \\
&\qquad\qquad\qquad\qquad\qquad\qquad \text{(Law of total probability)} \\
&= \frac{HR_k(X=0)h_k^0(X=0)Pr(X=0|Y_{k-1}^1=0) + HR_k(X=1)h_k^0(X=1)Pr(X=1|Y_{k-1}^1=0)}{h_k^0} \\
&= HR_k(X=0)\cdot\frac{h_k^0(X=0)Pr(X=0|Y_{k-1}^1=0)}{h_k^0} + HR_k(X=1)\cdot\frac{h_k^0(X=1)Pr(X=1|Y_{k-1}^1=0)}{h_k^0} \\
&= w_k(X=0)\cdot HR_k(X=0) + w_k(X=1)\cdot HR_k(X=1),
\end{aligned}
$$

where we denoted

$$w_k(X=0) = \frac{h_k^0(X=0)\cdot Pr(X=0|Y_{k-1}^1=0)}{h_k^0}$$

and

$$w_k(X=1) = \frac{h_k^0(X=1)\cdot Pr(X=1|Y_{k-1}^1=0)}{h_k^0}.$$

Then,

$$
\begin{aligned}
&w_k(X=0) + w_k(X=1) = 1 \\
\Leftrightarrow & h_k^0(X=0)\cdot Pr(X=0|Y_{k-1}^1=0) + h_k^0(X=1)\cdot Pr(X=1|Y_{k-1}^1=0) = h_k^0 \\
\Leftrightarrow & h_k^0(X=0)\cdot Pr(X=0|Y_{k-1}^1=0) + h_k^0(X=1)\cdot Pr(X=1|Y_{k-1}^1=0) = \\
& h_k^0(X=0)\cdot Pr(X=0|Y_{k-1}^0=0) + h_k^0(X=1)\cdot Pr(X=1|Y_{k-1}^0=0) \\
\Leftrightarrow & h_k^0(X=0)\left(Pr(X=0|Y_{k-1}^1=0) - Pr(X=0|Y_{k-1}^0=0)\right) = \\
& h_k^0(X=1)\left(-Pr(X=1|Y_{k-1}^1=0) + Pr(X=1|Y_{k-1}^0=0)\right) \\
\Leftrightarrow & h_k^0(X=0)\left(Pr(X=0|Y_{k-1}^1=0) - Pr(X=0|Y_{k-1}^0=0)\right) = \\
& h_k^0(X=1)\left(Pr(X=0|Y_{k-1}^1=0) - Pr(X=0|Y_{k-1}^0=0)\right) \\
\Leftrightarrow & \left(Pr(X=0|Y_{k-1}^1=0) - Pr(X=0|Y_{k-1}^0=0)\right)\cdot\left(h_k^0(X=1) - h_k^0(X=0)\right) = 0.
\end{aligned}
$$

References

[1] M. A. Hernán and J. M. Robins, *Causal Inference: What If.* boca raton: chapman & hall/crc. ed., 2020.

[2] J. Robins, “A new approach to causal inference in mortality studies with a sustained exposure period—application to control of the healthy worker survivor effect,” *Mathematical Modelling*, vol. 7, pp. 1393–1512, Jan. 1986.

[3] M. A. Hernán, “The Hazards of Hazard Ratios,” *Epidemiology (Cambridge, Mass.)*, vol. 21, pp. 13–15, Jan. 2010.

[4] R. A. J. Post, E. R. van den Heuvel, and H. Putter, “The built-in selection bias of hazard ratios formalized using structural causal models,” *Lifetime Data Analysis*, vol. 30, pp. 404–438, Apr. 2024.

[5] E. Shahar and D. J. Shahar, “More on Selection Bias,” *Epidemiology*, vol. 21, p. 429, May 2010.

[6] J.-C. Lin and W.-C. Lee, “Hazard Ratio Bias in Cohort Studies,” *Epidemiology*, vol. 24, p. 777, Sept. 2013.

[7] M. J. Stensrud, M. Valberg, K. Røysland, and O. O. Aalen, “Exploring Selection Bias by Causal Frailty Models: The Magnitude Matters,” *Epidemiology*, vol. 28, no. 3, pp. 379–386, 2017. Publisher: Lippincott Williams & Wilkins.

[8] M. Abrahamowicz, M.-E. Beauchamp, E. K. Roberts, and J. M. G. Taylor, “Revisiting the Hazards of Hazard Ratios Through Simulations and Case Studies,” *European Journal of Epidemiology*, May 2025.

[9] T. S. Richardson and J. M. Robins, “Single world intervention graphs (swigs): A unification of the counterfactual and graphical approaches to causality,” *Center for the Statistics and the Social Sciences, University of Washington Series. Working Paper*, vol. 128, no. 30, p. 2013, 2013.

[10] M. J. Stensrud and M. A. Hernán, “Why Test for Proportional Hazards?,” *JAMA*, vol. 323, pp. 1401–1402, Apr. 2020.

[11] M. J. Stensrud and M. A. Hernàn, “Invited Commentary: Why use methods that require proportional hazards?,” *American Journal of Epidemiology*, p. kwae361, Jan. 2025.

[12] S. Greenland, “Absence of confounding does not correspond to collapsibility of the rate ratio or rate difference,” *Epidemiology (Cambridge, Mass.)*, vol. 7, pp. 498–501, Sept. 1996.

[13] V. Didelez and M. J. Stensrud, “On the logic of collapsibility for causal effect measures,” *Biometrical Journal. Biometrische Zeitschrift*, vol. 64, pp. 235–242, Feb. 2022.

[14] Y. Liu, B. Wang, M. Yang, J. Hui, H. Xu, S. Kil, and J. C. Hsu, “Correct and logical causal inference for binary and time-to-event outcomes in randomized controlled trials,” *Biometrical Journal. Biometrische Zeitschrift*, vol. 64, pp. 198–224, Feb. 2022.

[15] A. Huitfeldt, M. J. Stensrud, and E. Suzuki, “On the collapsibility of measures of effect in the counterfactual causal framework,” *Emerging Themes in Epidemiology*, vol. 16, p. 1, Jan. 2019.

[16] A. Sjölander, E. Dahlqwist, and J. Zetterqvist, “A Note on the Noncollapsibility of Rate Differences and Rate Ratios,” *Epidemiology (Cambridge, Mass.)*, vol. 27, pp. 356–359, May 2016.

[17] R. Daniel, J. Zhang, and D. Farewell, “Making apples from oranges: Comparing noncollapsible effect estimators and their standard errors after adjustment for different covariate sets,” *Biometrical Journal*, vol. 63, no. 3, pp. 528–557, 2021. _eprint: https://onlinelibrary.wiley.com/doi/pdf/10.1002/bimj.201900297.

[18] D. R. Cox, “Regression Models and Life-Tables,” *Journal of the Royal Statistical Society. Series B (Methodological)*, vol. 34, no. 2, pp. 187–220, 1972. Publisher: [Royal Statistical Society, Oxford University Press].
